# *“In the army I’m no longer typecast as the son of migrant workers”*: modalities of inclusion and belonging among children of migrant workers in the military in Israel

**DOI:** 10.3389/fpsyg.2024.1300081

**Published:** 2024-01-24

**Authors:** Galia Sabar, Deby Babis, Anabel Lifszyc-Friedlander, Uzi Ben-Shalom

**Affiliations:** ^1^The Department of Middle Eastern and African History, Tel Aviv University, Tel Aviv, Israel; ^2^The Department of Sociology and Anthropology, Ariel University, Ariel, Israel; ^3^The Harry S. Truman Research Institute for the Advancement of Peace, The Hebrew University of Jerusalem, Jerusalem, Israel; ^4^School of Health Professions, Faculty of Medicine, Tel Aviv University, Tel Aviv, Israel; ^5^Gordon College of Education, Haifa, Israel

**Keywords:** children of labor migrants, army, belonging, inclusion, citizenship, Israel

## Abstract

This article explores how non-citizen minorities experience military service, focusing on children of international labor migrants who served in the Israel Defense Forces. During the 1990’s, Israel witnessed an influx of migrant workers, primarily from the Philippines, Latin America and Africa. However, due to Israeli immigration policies, neither they nor their Israeli-born children were eligible for citizenship. Consequently, upon reaching the age of 18, unlike their Israeli peers, these children were not recruited into the army. Furthermore, they lived under constant threat of deportation. Due to advocacy by civil society organizations, in 2006 and 2010 the government granted civic status (permanent residency) to approximately 1,500 children. This made them eligible for military service, a somewhat unique situation globally. Upon completion of their first year of military service, they were eligible for Israeli citizenship and their immediate family members were eligible for permanent residency. Through qualitative and quantitative research, we examine inclusion and belonging amongst children of migrant workers who served in the military. Our findings suggest that military service enabled them to overcome the exclusionary boundaries they experienced as children in three ways. Firstly, they achieved formal belonging by receiving citizenship. Secondly, they achieved informal belonging through the cultural and social capital this service accrued within Israeli society. Finally, for some, military service deepened their knowledge of Judaism and, in certain cases, led to conversion, thus fostering religious belonging. These three aspects facilitated inclusion and a sense of belonging for these formerly marginalized children while also enhancing their legitimacy within Israeli society. This unique case study contributes to ongoing global debates about the experiences of minority groups in the military.

## Introduction

In May 2022, First Lieutenant Kale and Sergeant Jessica were amongst 120 outstanding soldiers who received a prestigious award from the Israeli President on Israeli Independence Day. Kale and Jessica, unlike other soldiers who received this award, are children of migrant workers who joined the army despite not being Israeli citizens. Just a few years prior to receiving this prestigious honor, they lived on the margins of Israeli society subjected to discrimination and under constant threat of deportation from Israel. Kale and Jessica’s fear was shared by all children of migrant workers in Israel. However, following lengthy campaigns, in 2006 and 2010, some 1,500 children obtained permanent residency followed by citizenship (Willen, 2005; Kemp, 2007; Lifszyc-Friedlander, 2010; Babis et al., 2018; Herzog, 2023). Consequently, along with other Israeli citizens, they were expected to serve in the military^1^. This article will explore their transition from social marginalization, including discrimination and threats of deportation, to being drafted into the army along with most citizens in the State of Israel.

Globally, many host countries seek to prevent permanent settlement of labor migrants through policies which influence not only the migrants but also their children (Devasahayam, 2010; Shah, 2013). Such children, who are either themselves undocumented or have legal status while other members of their families do not, navigate their lives in territories of illegality (Fix and Zimmermann, 2001; Rodriguez, 2019; Terrazas et al., 2020). For children of migrant workers in most countries, joining the host country army is not feasible; therefore, little has been written about their experiences in the armed forces (Strader et al., 2021). This case study, focusing on children of undocumented migrant workers who joined the Israel Defense Forces (IDF), sheds new light on the significance of military service for marginalized groups and the modalities of inclusion and belonging these soldiers developed during their service.

Previous research has focused on the significance of receipt of civic status for children of migrant workers in Israel (Babis et al., 2018) and their experiences in and adaptation to their military service (Ben-Shalom et al., 2023). In this paper, we examine whether the discrimination and marginalization they faced as children of migrant workers continued during their time in the IDF. In other words, we deepen our understanding of modalities of belonging and inclusion in Israel through military service, following years of formal exclusion from the state in which they were born and/or raised. Therefore, this unique case study contributes to ongoing global debates about the experiences of minority groups in the military.

## Literature Review

### Immigration, military service, citizenship, and inclusion

Immigration, military service, citizenship, and inclusion are interrelated concepts with a long history. In the Roman Empire for example, non-citizens or peregrines [foreigners] were allowed to become naturalized by serving in the Roman army (Mathisen, 2006). In the United States, serving in the army opens the gates to citizenship for non-citizens, including undocumented migrants (Plascencia, 2015; Archer, 2022). Yet such military service does not offer automatic social inclusion or immunity from prejudice and discrimination.

While military service in most countries globally is not a prerequisite for obtaining civil status, military service, immigration and citizenship tend to be connected (Ware, 2010). Research has shown that, in some cases, military service contributes to social integration and the formation of national identity among immigrants (Mazumder, 2019). On-going exposure to state symbols such as the flag (Kalmoe and Gross, 2016), the official language and, of course, discourse related to defense of the homeland and the war effort (Yanagizawa-Drott, 2014), contribute to the transformation of immigrants into soldiers who want to fight for their new nation. Socialization can occur in the army because immigrants meet and interact with diverse populations and these interactions engender the formation of relationships between equals that would be hard to replicate elsewhere (Eisikovits, 2006). Such mechanisms of inculcation and socialization do not operate in a vacuum; they are related to factors specific to each country (macro-level), each military network (mezzo-level) and the immigrants themselves (micro-level) (Enloe, 1981; Ben-Shalom et al., 2023).

Furthermore, military service can open doors to immigrants that would otherwise remain closed (Lutz, 2008). It facilitates social mobility for immigrant populations who view military service as a source of sustenance, benefits and prestige (Trundle, 2012). It may also confirm that the immigrants’ host country also belongs to them (De Rosa and Szvircsev Tresch, 2022). Where army recruitment is voluntary, the decision to serve may be pragmatic stemming from a desire to acquire citizenship and overcome discriminatory experiences. It also may be a way to express gratitude to the country that welcomed them into its midst (Scoppio et al., 2022). Some argue that relationships established between diverse groups in the army increase tolerance in society at large (Mazumder, 2019).

### Children of migrant workers: exclusion, access to citizenship, and military service

Labor migration is a primary component of human migration in the 21st century. In 2020, the stock of international migrants was estimated to reach 281 million people; of these 169 million were labor migrants representing 60% of the total number (Black, 2021; ILO - International Labour Organization, 2021). Nation-states are challenged by the presence of labor migrants; they are not only workers, but human beings that bring their families with them or start families in host countries. Consequently, host countries develop different strategies to address their presence including making decisions about what civic status to grant children who arrive at an early age or who are born in the host country (Lifszyc-Friedlander, 2010; Hsieh, 2023).

States are presented with multiple and complex challenges around citizenship due to the presence of children of migrant workers (Bhabha, 2009; Paz, 2016). Some countries (such as Ireland, Denmark, Finland, Sweden, the Netherlands, and Italy) have made it more difficult for them to obtain civic status (Garner, 2007; Eurostat, 2010). Other countries (such as Germany, Portugal, Luxembourg and Greece) have relaxed legislation in this regard (Morje Howard, 2008; Matias, 2016). The United States and Canada apply the principle of Jus Solis meaning that children of migrant workers born in these countries receive citizenship upon birth. However, this does not change the undocumented status of their parents (Fix and Zimmermann, 2001).

Children of migrant workers who live in countries where the ability to acquire civic status is extremely limited or non-existent find themselves in an ambiguous situation. In many respects, they feel a sense of belonging to the host society because they attend local schools, learn the language and interact with native-born children, yet they lack civic status and the rights that this entails (Portes and Rumbaut, 2005; Meloni et al., 2014). Research has shown that legal exclusion generates trauma which may seriously affect the mental health of children of migrant workers. Many of them live in a state of fear and insecurity and experience helplessness, anxiety, feelings of incompetence, frustration and constant worry (Saad, 2013). These feelings usually appear during the later years of high school when children begin to fully comprehend that they are no longer protected by the state as minors rather have become vulnerable adults (Abrego and Gonzales, 2010; Hanson and Gonzales, 2011).

The relationship between the children’s status and army recruitment ranges from very inclusive to exclusive, depending on the country’s specific policies. In some countries, civic status is not a precondition to join the military; this is the case in Bahrain, Oman and the United States. The latter’s policy is the most inclusive; throughout history and up until the present time, the United States has not only recruited residents who are not citizens, but their incorporation into the military has expeditated their process of citizenship (Mazumder, 2019). In the continuum between inclusion and exclusion, there is a wide variety of criteria for recruitment of non-citizens. On the more inclusive side, Belgium and India recruit children of migrant workers who do not hold citizenship but are citizens of neighboring or allied countries. Australia and Canada recruit non-citizen migrants if they have specific skills such as doctors and pilots. Finally, countries such as Brazil, the Netherlands, Poland, Sweden and Switzerland engage in policies of exclusion; military recruitment is reserved for citizens only (Hanson and Lin-Greenberg, 2019; Greco and Scoppio, 2022). Yet, as mentioned earlier, military recruitment does not offer automatic inclusion or immunity from prejudice and discrimination either in the army itself nor within society at large.

## The case study

### Immigration, citizenship and military service in Israel

Israel defines itself as a Jewish state and a homeland for Jews worldwide. It grants citizenship based on the principal Jus Sanguinis meaning that descendants of Jews have the right to receive citizenship upon arrival in Israel. Once they reach the age of 18, they are expected to serve in the military along with other Israeli citizens as military service is compulsory for all Jewish populations groups in Israel.^1^ Both men and women are required to enlist.

Research demonstrates that the IDF is one of Israel’s most central and influential institutions. Army service is a primary symbol of belonging to the Israeli collective and plays a significant role in practices of civic inclusion (Sasson-Levy, 2002; Sasson-Levy, 2003; Eastwood, 2019). As Israel is an immigration country consisting of Jews of many different origins, military service facilitates an unmediated encounter between different populations. As such, it can be an opportunity - not always realized - for advancing and integrating immigrants (Azarya and Kimmerling, 1980; Shabtay, 1999; Ben-Shalom and Horenczyk, 2004; Dolberg and Amit, 2023).

Due to its centrality in Israeli life, the IDF has significant experience absorbing youngsters who come from very different social, cultural and economic backgrounds. Over time, it has developed very solid and detailed procedures to enable their service. The IDF is known as one of Israel’s most inclusive and diverse institutions; some claim that it has helped specific groups to overcome various forms of marginalization (Shabtay, 1999; Kanaaneh, 2005; Lomsky-Feder and Ben-Ari, 2013; Esensten, 2019). Other scholars, however, have pointed to socio-ethnic stratification in the army which not only reproduces class and ethnic inequalities and practices of exclusion, but also may legitimize Israel’s existing class-based social order (Levy, 1998; Levy and Sasson-Levy, 2008).

### Children of migrant Workers in Israel: exclusion, access to citizenship, and military service

Migrant workers began coming to Israel in the early 1990s. This was due to the first Palestinian uprising and repeated closures imposed on the Occupied Palestinian Territories which prevented Palestinian laborers from entering Israel. To meet the demand for manpower, the government imported migrant workers from abroad. This was the first time in Israel’s history that mass numbers of international migrant workers entered the country. Initially Israel allocated work permits primarily within the agriculture and construction sectors; later this was expanded to include live-in caregivers (Bar-Tzuri, 1996; Bartram, 1998). Within 10 years, Israel’s population of migrant workers reached around 240,000 (43% with valid work permits and 57% who were undocumented); they constituted 10% of Israel’s labor force. In spite of the experience gained in other western countries, Israel regards migrant workers as temporary. Various restrictive rules and regulations aimed at preventing them from settling in the country were issued including a ban on establishing families and raising their children in Israel. Hence, once international migrant workers gave birth in Israel their children were not eligible for Israeli citizenship (Shamir and Mundlak, 2013; Raijman and Kemp, 2016) and their parents had either to send them home or, if opted to raise them in Israel, they lost their visas and hence their stay became illegal. In spite of these regulations, over the years thousands of children were born to international migrant workers (Lifszyc-Friedlander, 2010; Kemp and Kfir, 2016). Although they had no civic status, they are eligible to enter the Israeli educational system and do enjoy some social rights. Yet, despite growing up and studying in Israeli schools, absorbing its culture and speaking fluent Hebrew (Eliyahu-Levi and Ganz-Meishar, 2016; Winer et al., 2021; Tannenbaum and Stern, 2022), their children are pushed to the margins of society. They suffer from various forms of formal and informal discrimination, are not eligible for civic status and become candidates for deportation (Willen, 2005; Sabar and Gez, 2009).

In 2001, the State of Israel began deporting undocumented migrant workers and their families. In order to prevent deportation of their children, local and international civil society organizations waged campaigns seeking to achieve civic status for them. Following many difficult years of advocacy, in 2006 the government decided to grant permanent residency to children of migrant workers who fell within specific criteria. This was a one-time humanitarian gesture that was subsequently repeated in 2010. Consequently, around 1,500^2^ children of migrant workers were eligible for permanent residency in Israel with their parents and siblings receiving temporary residency. While temporary residency required renewal on an annual basis, it enabled them to obtain legal work permits (Willen, 2005; Kemp, 2007; Sabar and Gez, 2009; Kemp and Kfir, 2016; Babis et al., 2018). The government stipulated that following completion of their first year of military service, children of migrant workers would be eligible for citizenship and their parents and siblings would be eligible for permanent residency (Lifszyc-Friedlander, 2010; Herzog, 2023).

The research presented here focuses on modalities of belonging to the State of Israel and Israeli society which children of migrant workers acquired during their military service. We explore whether their experiences of discrimination and marginality as children of migrant workers continued within the IDF. In other words, has formal inclusion via the ability to serve in the IDF enabled them to overcome forms of exclusion from the state in which they were born and/or raised?

## Methodology

This mixed methods study combines quantitative and qualitative research enabling us to capture a holistic view of the phenomenon of children of migrant workers who joined the IDF and their experiences of service. Quantitative data was collected in 2020 through a digital survey completed by 154 participants. The questionnaire, written in Hebrew, covered different aspects of children’s experiences including their general feelings regarding military service, their relationship with their commanders and with other soldiers, their involvement in military operations, and an open-ended question on possible benefits of their time in the IDF. The questionnaire was shared via WhatsApp with several children of migrant workers already known to the researchers. They, in turn, helped to further distribute the questionnaire by providing names and phone numbers of relevant additional participants. They also shared the survey in their WhatsApp groups. The only criteria for participation in the survey was that the respondents must be children of migrant workers who had served in the IDF. Participation was voluntary and anonymous.

We applied quota sampling leading to respondents of the demographics outlined here. In terms of gender, 53% were female and 47% were male. All age groups were represented as follows: 18–21 years old - 40%; 22–25 years old - 37%; 26–35 years old - 23%. Different communities of migrant workers in Israel were represented as well including: 51% Asian (The Philippines, Thailand), 27% African (Nigeria, Ghana, Congo, Kenya) and 22% Latin American (Colombia, Peru, Ecuador). The sample also reflected the geographical distribution of children of migrant workers in Israel: 56% of the participants live in Tel Aviv and 44% were from outside of Tel Aviv.

Qualitative data were collected by means of open-ended questions in the quantitative digital survey, digital ethnography and semi-structured interviews. Interviews took place during two different periods: between 2015–2016 and in 2019–2020. In total, we interviewed 26 individuals: 14 male and 12 female between the ages of 18–30. Of these, six have parents from Latin America, seven have African parents and 13 are children of people from the Philippines and Thailand. The interviews were conducted in Hebrew and lasted between 40 min and 3 hours. Questions focused on different experiences during service, their feelings and their insights in retrospect. Additional qualitative data was collected by means of digital ethnography on Facebook between 2015 and 2022; this data involved analyzing the Facebook walls of children of migrant workers and their parents. Access to Facebook was possible because one of the authors has a wide network of Facebook friends comprised of migrant workers and their children, all of whom were aware that this author is a researcher.

Quantitative data was analyzed using standard statistical tools while the qualitative data was analyzed in accordance with the principles of grounded theory (Strauss and Corbin, 1994). We present the findings in an integrated way which allows us to simultaneously introduce different dimensions of the phenomenon. For ethical reasons, names cited in the paper have been changed to respect participant anonymity and protect their privacy.

## Findings

Following an overview of the findings regarding the IDF service of children of migrant workers, we focus on their perceptions of inclusion and exclusion from the IDF. This is expressed in the modalities of belonging that they acquired and developed during their military service.

**Figure 1:**
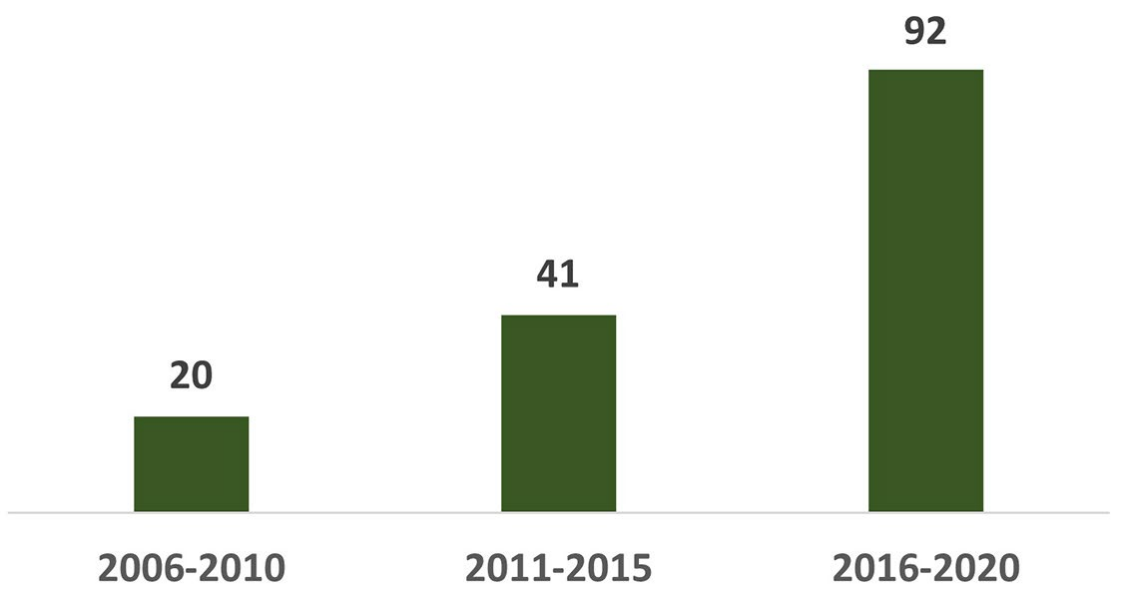
Enrolment of children of migrant laborers to the IDF by years (*N* = 153).

Since 2006 when Israel first granted civic status to children of migrant workers, they began to be recruited into the army. According to our sample, their numbers have risen over the years (See Figure 1).^3^

Our findings indicate that the research population was highly motivated to join the IDF. Their years in service were perceived as being very positive and represented a great source of pride for them. Yet it was clear that, unlike other Israeli youngsters, their motivation also stemmed from their unique life experiences growing up on the margins of society where they were exposed to various forms of exclusion mainly due to their lack of civic status. Joy, for example, spoke about her reasons for joining the army; she emphasized her gratitude to the country that enabled her to feel included:

I really wanted to join the army …. There are those [native Israelis] who try to avoid the army, but I did not, I wanted … to represent my country with honor … to thank the country that eventually accepted me. -Joy, 26 years-old

Juan talked about his desire to prove himself worthy of being part of the Israeli nation and society:

By serving in the IDF, I proved to myself and my country that I’m like all other citizens who grew up in Israel. -Juan, 24 years-old

As with many other armies globally, service in the IDF is divided into two main categories: combat and non-combat units. Joining combat units is perceived as being more prestigious for both male and female soldiers. It can require not only high levels of motivation and physical abilities but also a rigorous selection process. Our data show that 51% of males and 20% of females were enrolled in combat units. Tonny, 22, said:

Nowadays many youngsters want to have an easy job in the IDF …. We, the Filipinos, are different … we want to be in combat units..otherwise it does not count.

Altogether, 95% of our respondents said that they were proud of their service in the IDF; they stressed that it was not only a duty but also an honor and part and parcel of what it means to be Israeli.

### Becoming Israeli: modalities of inclusion and belonging acquired during military service

Our digital survey concluded with an open-ended question asking: “What, if at all, has your IDF service contributed to you?” Participant responses to this question demonstrate that serving in the IDF not only enhanced their personal development (e.g., gave them self-confidence, prepared them for life) but also expanded their sense of inclusion and strengthened the Israeli parts of their identity or, what we termed, their ‘Israeliness.’ This was first expressed as a self-signifier: *“I did my IDF service like any other Israeli and I have the same rights,”* and subsequently as an indication of external recognition: “*Being a soldier allowed me to be formally recognized as an Israeli” and “I’m now part of the Israeli nation.”* These findings are also reflected in the quantitative data; 82% of those who did not feel ‘totally Israeli’ prior to serving in the IDF (65% of the sample) felt that the IDF completed their process of becoming Israeli. Indeed, we found that serving in the IDF offered children of migrant workers a much desired sense of inclusion via an ‘entry ticket’ into three major dimensions of Israeliness: *formal belonging, informal belonging* and *religious belonging.*

### Formal inclusion and belonging

The Israeli government decision of 2006 established that following completion of their first year of military service, children of migrant workers would be eligible for citizenship. Military service not only grants them a legal path to Israeli citizenship and consequently an Israeli passport but also gives them the option of taking political action as it includes the right to vote. The official occasion where they are asked to pledge allegiance to the State of Israel is documented and celebrated by them and members of their family. For example Mary, whose son served in the army, posted pictures of the citizenship ceremony writing: *“Congratulations son. Daddy, mommy and the whole family cannot be more proud.”* Another proud mother posted: *“You have got it …*. *You are now truly an Israeli citizen, Mazal Tov [congratulation in Hebrew] - we are so happy for you.”*

Serving in the IDF also provides the children’s parents and siblings with the right to apply for permanent residency, and eventually citizenship. Sheyla, a 23-year-old Filipina soldier said:

One of the reasons, but not the only one, to join the IDF is to gain quick access to citizenship …. I’m my family’s anchor because thanks to me they will all get permanent residency …. Their fear of being denied [status] is over.

Obviously Sheyla was referring to denial of status that she and her family had endured for years. However, it also signifies a more general denial of their rights, hopes and recognition – indeed, the desire to be like those who are included rather than excluded. Furthermore, IDF service brings with it the possibility of family reunification as they can request entry into Israel of family members who were previously deported. Jenny, a 29 year-old woman whose father was deported to Thailand when she was 10 years old and remained in Israel with her Filipina mother said:

I joined the IDF … and as a soldier I discovered that there is a procedure that enables soldiers to invite their parents to Israel as permanent residents … so I filed a request and it was denied …. I filed again and it was denied …. Only the third time, after seven years, it was approved …. My father is in Israel now.

### Informal inclusion and belonging

**Figure 2:**
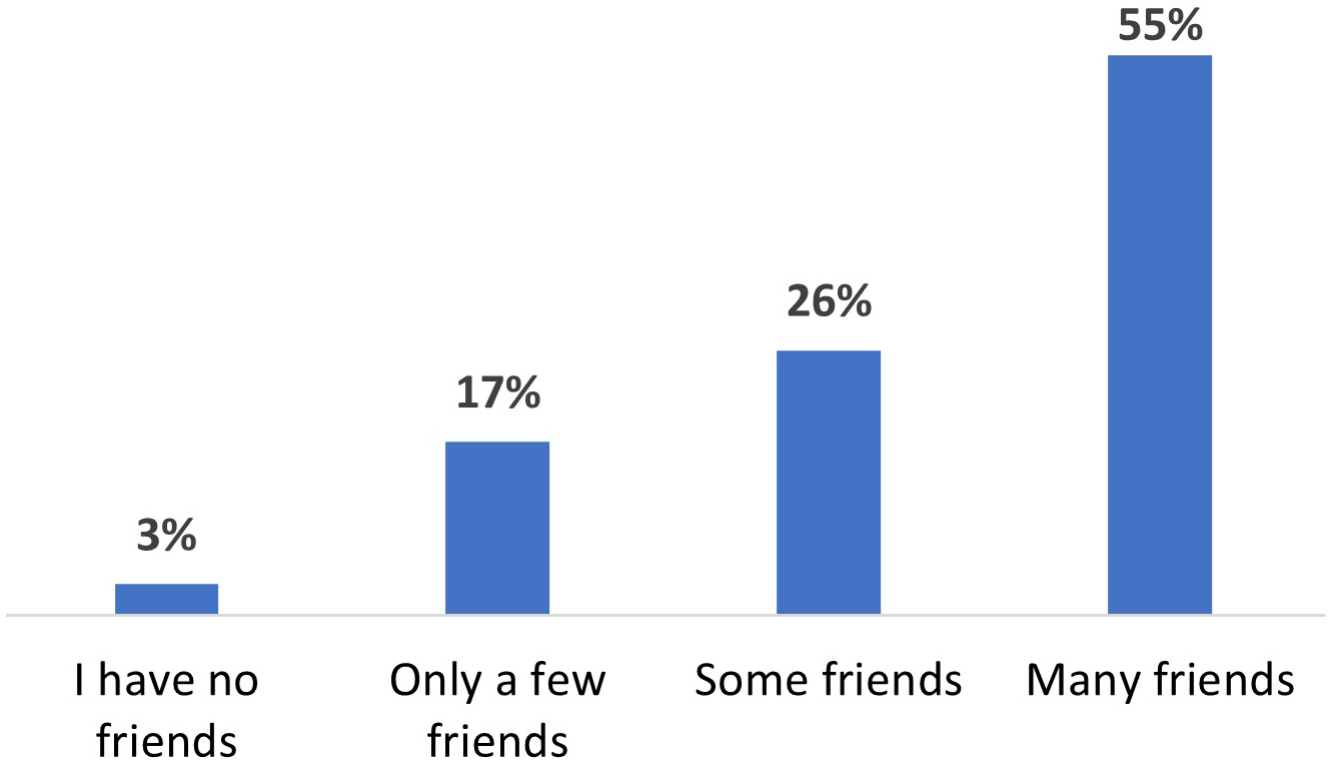
To what extent did you make friends while serving in the IDF? (*N* = 154).

Beyond formal belonging, serving in the IDF expanded informal levels of inclusion and belonging, primarily because it enhanced their social and cultural capital. In our quantitative survey we asked: “*To what extent did you make friends while serving in the IDF?”* As can be seen in Figure 2, the vast majority (97%) acquired new friends in the army, hence expanding and strengthening their Israeli social network.

**Figure 3:**
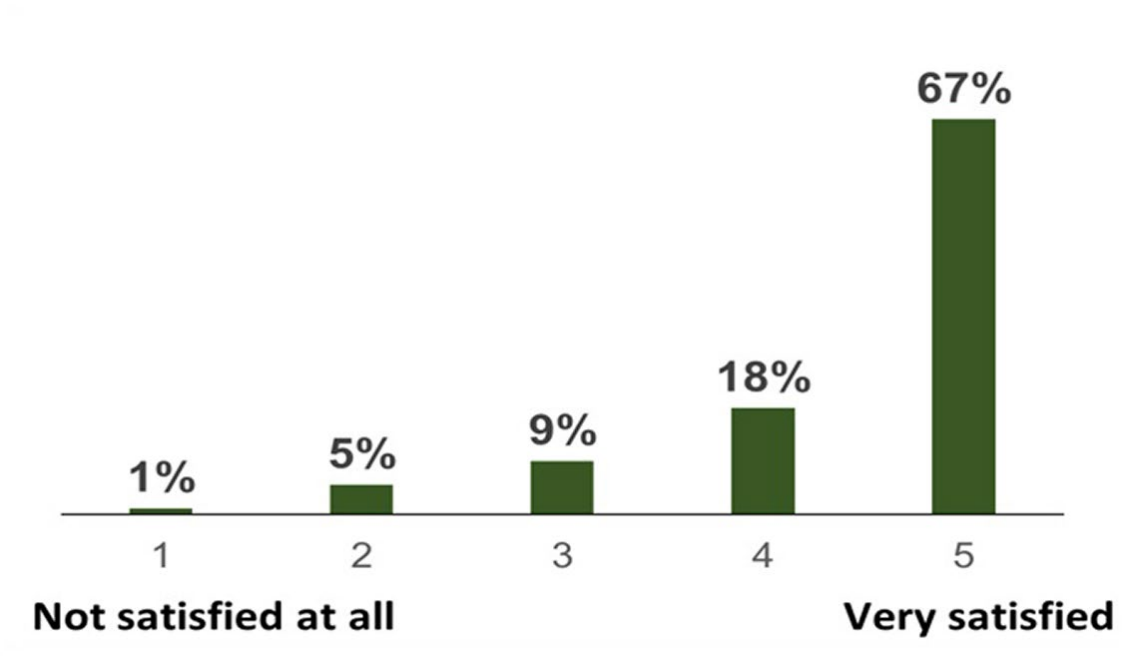
To what extent are you content with your social ties with other soldiers in your unit? (*N* = 154).

Not only did the number of their friends increase, but the majority (85%) were very happy with newly developed social ties through their units as can be seen in Figure 3.

This feeling of inclusion was also reiterated on Facebook posts where children of migrant workers shared pictures of themselves with their friends in the army. For example, V. posted a picture with two of his friends from his unit saying: *“Shabbat Shalom [Happy weekend] with my amazing brothers.”* The importance of these relationships was also mentioned in our digital survey; in the open-ended question some participants noted that this was the most significant element of their military service saying: *“I wanted to make friends that are not children of other migrant workers but Israelis … true ones, who live here … to know how they feel as Israelis who live here.”*

Beyond expanding their social networks, some mentioned that during their service they became acquainted with new places, new cultures and new modalities of life in Israel by visiting their newly acquired friends. One said to us:

In the IDF I came to know the country … towns I did not know … kibbutzim and others …. During our free time in the IDF we used to go to sit in bars all over Israel … or just roam around in Jerusalem …. You know, these regular things …. Now when we are out of the IDF … we go to visit friends all around the country. -Sonny, 27 years-old

**Figure 4:**
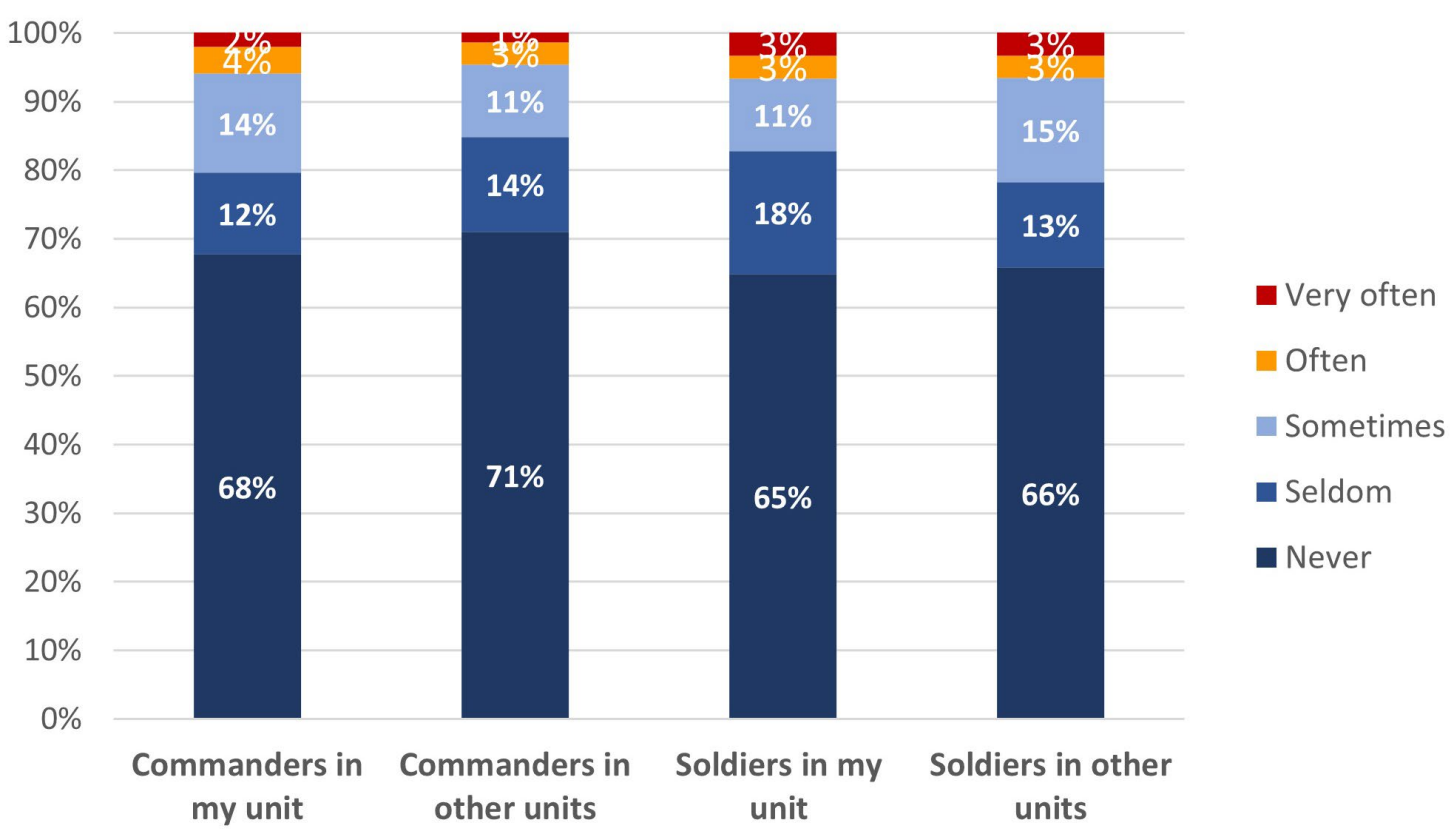
How often, if at all, have these people in the army treated you unfairly because you are a child of migrant laborers?

One of the most significant research findings was that during service our informants rarely encountered unfairness, prejudice or stereotypes related to migrant workers. This stands in sharp contrast with overall negative attitudes, unfairness and violent practices toward migrant workers in Israel (Raijman and Semyonov, 2004). Figure 4 indicates that in response to the question: *How often, if at all, have different people in the army treated you unfairly because you are a child of migrant workers?,* respondents indicated that only rarely did commanders or low-ranking soldiers practice these malicious behaviors.

Moreover, many emphasized that, for the first time in their lives, they were free of the label ‘children of migrant workers.’ Rather were considered to be soldiers just like everyone else. Richard, 23 years-old, said:

You enter the IDF and there is no category: ‘son of migrant workers.’ This is an amazing feeling … they all treated me like any other soldier. In the IDF it does not matter who you are, all people are equal.

This experience was only possible within the military habitus as the military space enhances a sense of togetherness. Moreover, by its very nature, the military is based on personal achievements and motivation rather than on pre-existing socio-economic and other categories. Hence, their military service freed them from the category they carried all their lives: children of migrant workers.

Notwithstanding, one of our interviewees did mention that some soldiers held erroneous notions about Africans and about non-Jewish people. They only changed their mind about this following service in the same unit with him. He said:

Some people have an opinion about blacks … but when they got to know me they realized that not all blacks and not all Christians are murders. -Elias, 22 years-old

Rosalie clearly stated that within the IDF she did not encounter prejudice and did not feel excluded saying: “*I have not experienced racism in the IDF.”* Yet, later on in the interview she gave examples of remarks that could be interpreted as racist or prejudiced but she characterized them as ‘stupidity’ saying: “*I did, however, encounter stupid remarks from ignorant people who do not know that in Israel there is a generation of children of migrant workers … like me. -Rosalie, 26 years-old.*

Finally, within the informal level of inclusion and belonging, IDF soldiers received recognition and appreciation from various members of Israeli society beyond the army. Daniel said:

Everywhere I went [with my IDF uniform] people saluted me and thanked me …. Even when I hitchhiked people would say: ‘where are you from’ and then when I told them they would salute me and say, ‘woohooo this is amazing what you have given to this country, you are one of us. you are a sabra [native born Israeli]. Bravo.’ -Daniel, 23 years-old

Based on her life experiences as the daughter of persecuted undocumented migrant workers, public recognition as a worthy member of Israeli society was both somewhat unexpected and also very gratifying to Laila. She said:

I used to walk around in my IDF uniform and out of nowhere people would stop me and salute me …. It was weird …. I don’t know if they did it because they thought I was in a combat unit or because I am a Filipina. -Laila, 24 years-old

### Religious inclusion and belonging

**Figure 5:**
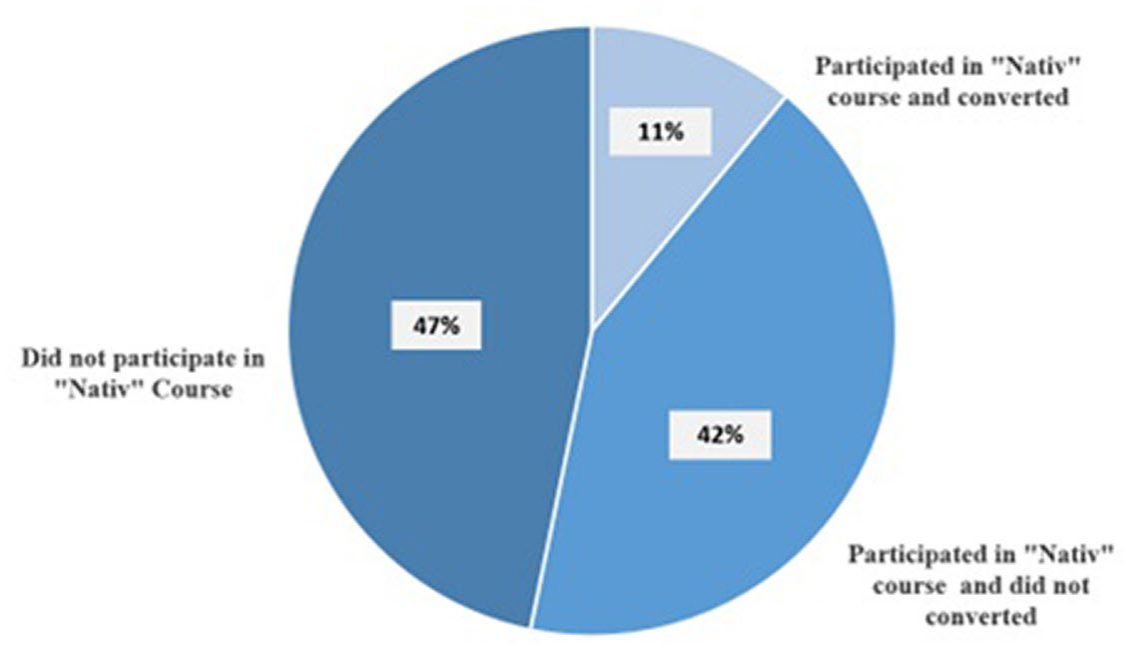
Participation and conversion in the “Nativ” course in the army (*N* = 154).

The third and final level of inclusion and belonging which was enhanced through military service is Jewish belonging. The IDF offers a conversion course called *Nativ*^4^ to children of migrant workers. It includes topics related to Judaism including Jewish rituals, rules and regulations as well as information on the history and sociology of Israel. Our findings revealed that 11% of those who participated in *Nativ* during their military service converted to Judaism (See Figure 5). Robby, who joined the course and converted said:

When I joined the IDF they told me to join a conversion course …. I went … you meet many that are like me, not the same color but not Jewish …. In the course you need to decide if you want to go through conversion or not …. You get to travel all over the Holy Land and then when you finish you need to observe religious practices … take a test and then you become a Jew. -Robby, 25 years-old

David, who also participated and completed the conversion process said:

This whole conversion came because I grew up in Israel and I wanted to be connected to Judaism …. As a child I was invited to my Israeli friends for Shabbat [Sabbath] dinners …. I liked the togetherness of Jews in the holidays …. Today I’m a ‘kosher’ Israeli and a Jew …. BUT [I can’t forget that] for ten years I was considered to be the son of migrant workers … with no rights, nothing. -David, 27 years-old

As noted, only 11% actually converted^5^ however from data collected via interviews amongst those who participated but did not convert (42%), many indicated that the course offered them the opportunity to deepen their knowledge about Judaism and other topics that are relevant for them as Israelis. Furthermore, despite having not converted, many felt included within the Jewish peoplehood as a result of the course. Camila said:

Unfortunately, I did not manage to convert… I did some of the prayers and rituals practiced in Judaism … but … I’m more Jewish and Israeli than half of the Israelis who do not keep the commandments. -Camila, 24 years-old

It should be noted that this final component of their identity is complex primarily because these soldiers are connected to extended family members who were not involved in this process.

## Discussion - from exclusion to inclusion via military service

The inclusion of children of migrant workers in the Israeli military was one of the outcomes of the campaign for the rights of children who grew up in Israel as part of an excluded and segregated population living under fear of deportation. Upon being granted permanent residency, military service became an option for them in the same way that it is open to other Israelis. Based on an analysis of data presented here, we claim that children of migrant workers who served in the IDF received an ‘entry ticket’ into Israeli society. This ‘entry ticket’ consisted of three primary dimensions: formal level of inclusion and belonging, informal level of inclusion and belonging and religious level of inclusion and belonging. Each dimension on its own and the juxtaposition of all three together created their unique sense of ‘Israeliness.’ Moreover, the feeling of being included in Israel was, for many, a new experience as throughout most of their lives they were excluded and suffered from various forms of racism and prejudice. Yet within the IDF, they rarely encountered the unfairness, prejudice or stereotypes commonly experienced by migrant workers.

At the *formal level of inclusion and belonging,* military service enabled children of migrant workers to apply for citizenship and, consequently, their immediate family members were eligible to apply for permanent residency. Receiving Israeli passports, the outcome of their status as citizens, enabled them free movement globally and established the legal inclusion of their families as well as protecting them from deportation. At the *informal level of inclusion and belonging* the army expanded the depth and breadth of their social ties. During their military service, they formed relationships with many other soldiers who came from different sectors of Israeli society. These newly forged and meaningful friendships both enriched their own individual social capital and expanded their knowledge of Israeli culture and physical geography. It also enabled them to pro-actively engage in attempts to change existing deeply-rooted stereotypes in Israel toward migrant workers and eradicated their previous identifier: children of migrant workers. In some ways, this finding corresponds with research by Mazumder (2019) who claimed that relationships established between diverse groups in the army increases tolerance in society at large, especially toward minority groups. Finally, at the *religious level of inclusion and belonging*, participation in the conversion course gave children of migrant workers the opportunity to expand their knowledge about Judaism, Israeli society, and Israeli culture and history regardless of whether they converted or not. Notwithstanding the difficulties, serving in the IDF provided them with formal recognition of their inclusion into the State of Israel and Israeli society.

As mentioned above, in other historical and contemporary contexts, army service is a known mechanism of integration (Chang, 1986; Szvircsev Tresch and Sokoli, 2013; Daithota Bhat, 2021) and, at times, is a central vehicle for gaining citizenship in countries like the United States (Plascencia, 2015; Ponti, 2018; Chishti et al., 2019). However, in Israel these processes are even stronger due to the special place the IDF occupies in Israel’s national and social consciousness. To date, most research on the IDF as a mechanism for integration and mobilization has focused on Jewish immigrants to Israel (Ben-Shalom and Horenczyk, 2004; Lomsky-Feder and Ben-Ari, 2013). These researchers and others emphasize the role of military service as a channel for social integration and mobility for those immigrants (Azarya and Kimmerling, 1980; Shabtay, 1999; Ben-Shalom and Horenczyk, 2004). Dolberg and Amit (2023), for example, claim that military service represented a turning point in the lives of young adults in the 1.5 generation as it provided them with familiarity with Hebrew and Israeli culture and society and gave them a sense of belonging to Israel and Israeli identity. However, to date, research in Israel has not focused on soldiers who were born to non-Jewish migrant workers and, due to their unique circumstances, were subsequently able to join the IDF. While Jewish immigrants entered the army with limited knowledge of Hebrew and Israeli culture and society yet as Israeli citizens, children of migrant workers’ situation was the opposite. They spoke Hebrew, had a good understanding of Israeli society and culture – despite living on the margins of Israeli society and experiencing racism and fear of deportation – yet were excluded from Israel on the official level due to their lack of civic status. Military service enabled them to mostly break free of these boundaries of exclusion. It fortified their status, enhanced their legitimacy within Israeli society and contributed to a more inclusive definition of who is Israeli. In other words, the definition of who is Israeli expanded not because of institutionalized processes, rather instead due to soldiers who are children of migrant workers.

Our study contributes to ongoing global debates about military service for non-citizen minorities. In order to further deepen understanding of the role of military service in Israel as it relates to processes of identity formation and social integration of minority groups, we suggest comparing our data to the experiences of other non-Jewish minorities (all of them Israeli citizens) such as Druze, Christian Arabs, Circassians, Muslims and Black Hebrews (known also as Afro-American Hebrew Israelites) serving in the IDF.

## Epilog

After many years of navigating their lives in Israel in zones of illegality and exclusion, children of migrant workers have become an integral part of the Israeli people and Israeli society not only *de facto* but *de jure*. Moreover, serving in the IDF empowered them to fight on behalf of the voiceless children of migrant workers who continue to suffer from racism and prejudice, those who have yet to receive civic status and are at risk of deportation. In other words, military service enabled them to become proud, loyal citizens. They are activists who feel secure enough and strong enough in their position to demand a change in Israeli attitudes and state policies toward children of undocumented migrant workers. In 2019, in the midst of a major deportation operation, they wrote a letter which was sent to the Prime Minister, the Minister of Interior and the President of Israel. It, in part, said:

We write you now, not as children but as adults who have served in the Israeli military with love and pride, who were once in the same situation as these children that you are trying to arrest and deport now. We too, like the children of migrant workers today, were born in Israel, were educated in Israeli schools, and we too were marked for deportation… We grew up and served in the military, we got married and some of us have become parents of young children. We are model citizens and we are living proof that Israel can only gain by not deporting these children.

## Data availability statement

The raw data supporting the conclusions of this article will be made available by the authors, without undue reservation.

## Ethics statement

The studies involving humans were approved by the Ethics committee at Ariel University. The studies were conducted in accordance with the local legislation and institutional requirements. The participants provided their written informed consent to participate in this study.

## Author contributions

GS: Conceptualization, Investigation, Project administration, Supervision, Writing – original draft, Writing – review & editing, Methodology. DB: Conceptualization, Formal analysis, Investigation, Methodology, Writing – original draft, Writing – review & editing. AL-F: Methodology, Writing – original draft. UB-S: Project administration, Writing – review & editing, Methodology.

## Footnotes

^1^Military service is compulsory with a few exceptions. The most notable and controversial is the exemption granted to Ultra-Orthodox Jews. However, recently some Ultra-Orthodox join the army though in small numbers (Malovicki-Yaffe et al., 2023). It should be noted that military service is also compulsory for the Druze minority however for Bedouins and other Arab citizens of the country, military service is not compulsory, yet individuals can choose to volunteer.

^2^The number 1500 is based on various estimates, as the Israeli government never released definitive statistics on the number of children of migrant workers who were granted civic status.

^3^It should be noted that there is no official data as to how many children of migrant laborers were drafted to the IDF.

^4^The *Nativ* course was originally created for immigrants from the Former Soviet Union that came to Israel under the Law of Return (which enables Jewish people and their descendants to immigrate to Israel) but who were not considered to be Jewish according to Jewish religious law. When these immigrants or their children join the IDF, the army offers them a course about Judaism, called Nativ [Path] which enables them to convert to Judaism. This course is offered by the army because it perceives itself to be an agent of integration into Israeli society. Some opt to convert while others do not. To the best of our knowledge, the IDF does not release data on the number of children of migrant workers who have participated in *Nativ* courses, hence, all of our insights are based on data that we collected in our study.

^5^While it is beyond the scope of this paper to address the low numbers of converts, two primary reasons explain this. The first relates to some participants’ reluctance to convert while the second is due to barriers erected by the Orthodox Jewish establishment which has the final say in the conversion process.
